# Identification of novel genetic regulations associated with airway epithelial homeostasis using next-generation sequencing data and bioinformatics approaches

**DOI:** 10.18632/oncotarget.19752

**Published:** 2017-07-31

**Authors:** Chau-Chyun Sheu, Ming-Ju Tsai, Feng-Wei Chen, Kuo-Feng Chang, Wei-An Chang, Inn-Wen Chong, Po-Lin Kuo, Ya-Ling Hsu

**Affiliations:** ^1^ Graduate Institute of Clinical Medicine, College of Medicine, Kaohsiung Medical University, Kaohsiung, Taiwan; ^2^ Division of Pulmonary and Critical Care Medicine, Department of Internal Medicine, Kaohsiung Medical University Hospital, Kaohsiung, Taiwan; ^3^ Department of Internal Medicine, School of Medicine, College of Medicine, Kaohsiung Medical University, Kaohsiung, Taiwan; ^4^ Welgene Biotech Inc, Taipei, Taiwan; ^5^ Department of Respiratory Therapy, College of Medicine, Kaohsiung Medical University, Kaohsiung, Taiwan; ^6^ Center for Biomarkers and Biotech Drugs, Kaohsiung Medical University, Kaohsiung, Taiwan; ^7^ Graduate Institute of Medicine, College of Medicine, Kaohsiung Medical University, Kaohsiung, Taiwan

**Keywords:** next-generation sequencing, bioinformatics, microRNA, airway remodeling, asthma

## Abstract

Airway epithelial cells play important roles in airway remodeling. Understanding gene regulations in airway epithelial homeostasis may provide new insights into pathogenesis and treatment of asthma. This study aimed to combine gene expression (GE) microarray, next generation sequencing (NGS), and bioinformatics to explore genetic regulations associated with airway epithelial homeostasis. We analyzed expression profiles of mRNAs (GE microarray) and microRNAs (NGS) in normal and asthmatic bronchial epithelial cells, and identified 9 genes with potential microRNA-mRNA interactions. Of these 9 dysregulated genes, downregulation of *MEF2C* and *MDGA1* were validated in a representative microarray (GSE43696) from the gene expression omnibus (GEO) database. Our findings suggested that upregulated mir-203a may repress *MEF2C*, a transcription factor, leading to decreased cellular proliferation. In addition, upregulated mir-3065-3p may repress *MDGA1*, a cell membrane anchor protein, resulting in suppression of cell-cell adhesion. We also found that *KCNJ2*, a potassium channel, was downregulated in severe asthma and may promote epithelial cell apoptosis. We proposed that aberrant regulations of mir-203a-*MEF2C* and mir-3065-3p-*MDGA1*, as well as downregulation of *KCNJ2*, play important roles in airway epithelial homeostasis in asthma. These findings provide new perspectives on diagnostic or therapeutic strategies targeting bronchial epithelium for asthma. The approach in this study also provides a new aspect of studying asthma.

## INTRODUCTION

Asthma is a chronic inflammatory and obstructive airway disease caused by the combination of genetic inheritance and environmental stimuli [1]. The inflammation in asthma airways initiates from the exposure of bronchial epithelium to environmental stimuli, leading to activation of immune cells and secretion of cytokines and chemokines [2, 3]. The dysregulated inflammatory mediators in airway wall leads to epithelial cells detachment or shedding [4], thickened mucosa [5], smooth muscle contraction and hypertrophy [6], increased vascularity [7], and abnormal deposit of collagen in subepithelium. The process of structural change and hyperresponsiveness in airway wall is called “airway remodeling” [8]. Airway remodeling is associated with the persistence of asthma symptoms and poor clinical outcomes [9]. The perturbation of bronchial epithelial homeostasis is considered to play a vital role in airway remodeling [10]. Bronchial epithelial cells function as a front-line barrier to respond to environmental stimuli and thus are susceptible to be constantly affected by inflammatory factors and physical injury. The damaged epithelial cells are then cleaned by immune cells or detached from the basement membrane [11–13]. It was demonstrated that the damaged epithelial cells undergo apoptotic programmed cell death, so the surviving or newly differentiated epithelial cells can proliferate to maintain tissue integrity [14, 15]. Higher rates of bronchial epithelial cell apoptosis have been identified in asthmatic patients than in healthy persons [16]. In addition, studies suggested that cellular autophagy and necrosis are involved in the damaged epithelial cells as well [17–20]. We also found that certain environmental pollutants, with estrogen-like activities can promote apoptosis in human bronchial epithelial cells [21]. These evidence provides a concept of which bronchial epithelial cells in asthmatic patients are more sensitive or susceptible to external environment, and this phenomenon may be regulated by a cohort of genetic networks related to cellular proliferation or homeostasis.

MicroRNAs are a group of small molecules containing 20-26 nucleotides, which can powerfully regulate gene expression by post-transcriptional modification. The regulatory mechanism is via the binding of microRNAs to the 3' untranslated region (3' UTR) of corresponding specific messenger RNAs (mRNAs), leading to degradation or translation inhibition of target mRNAs [22–24]. The amount of microRNAs is delicately regulated, and in addition, the targets of microRNAs may also affect the expression of other microRNAs [25]. The cooperative regulatory networks implicates that microRNAs can serve as either downstream or upstream effectors involved in one or many signaling pathways in response to cellular biological processes. The important roles of microRNAs have been highlighted and widely investigated in many diseases, particularly cancers [25]. In epithelial cells, the effects of microRNAs have been reported to be associated with a variety of fundamental functions, including proliferation, differentiation, and apoptosis [26, 27]. In a study using asthma as disease model, dysregulated expression of microRNAs affects bronchial epithelial cells growth and secretion of inflammatory factors in asthmatic biopsy [28].

Next-generation sequencing (NGS) is a powerful technique being applied to analyze the whole genome profile, including mRNAs and small RNAs expression, DNA copy number, DNA methylation, nucleotide structural variations, and transcription factor binding sites [29]. So far, this high-throughput profiling technology has discovered that numerous genes or microRNAs are critical effects of therapeutic targets in certain diseases [30–34]. In this study, we combined microarray and NGS analysis and sought to identify the potential microRNAs-mRNAs interactions in asthmatic bronchial epithelial cells. To further systematically analyze the candidate genes and microRNAs, several bioinformatics databases were used, including miRmap, DAVID, and GEO. Briefly, miRmap can be used to predict putative targets of microRNAs [35], and the Database for Annotation, Visualization and Integrated Discovery (DAVID) can classify a list of interesting genes into clusters by functional annotation [36]. To elevate the impact of the potential targets identified in asthmatic bronchial epithelial cells, we analyzed gene microarray of asthmatic patients from Gene Expression Omnibus (GEO) database [37].

There is neither cure against asthma nor effective biomarkers for diagnosis nowadays. This study aimed to identify potential microRNAs-mRNA interactions regulating the homeostasis of asthmatic bronchial epithelial cells. Hopefully the approach and findings of this study will provide new perspectives on the development of diagnostic or therapeutic strategies for asthma.

## RESULTS

### Identification of potential microRNA-mRNA interactions in asthmatic bronchial epithelial cells

**Figure 1:**
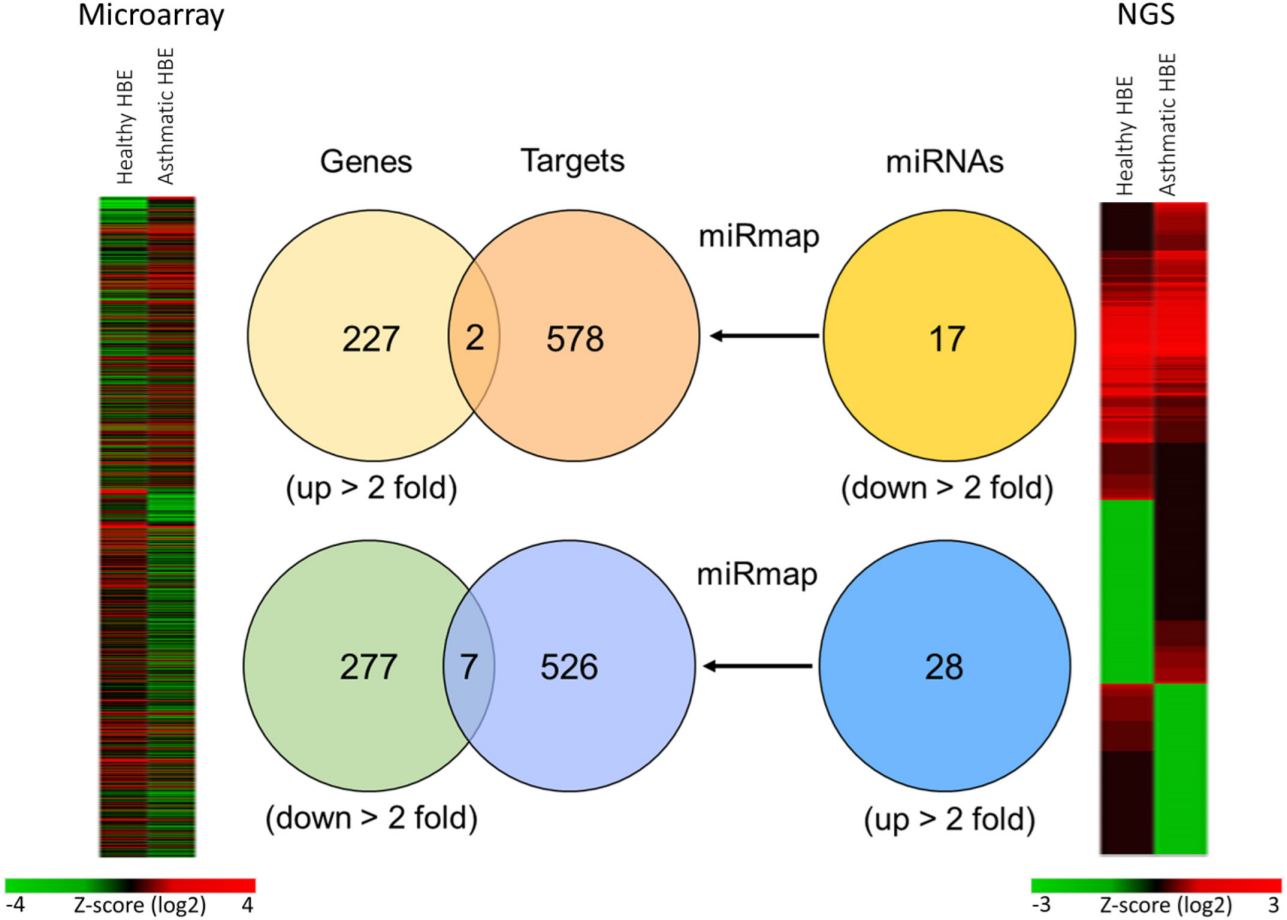
Representative diagrams of identification of potential microRNA-mRNA interactions in asthmatic bronchial epithelial cells GE microarray heatmap (left) analysis showed differentially expressed genes (total 504 genes) with fold change > 2. The “Genes” Venn diagram showed that 227 genes were upregulated and 277 genes were downregulated in asthmatic bronchial epithelial cells compared to normal bronchial epithelial cells. Next generation sequencing (NGS) heatmap (right) analysis showed differentially expressed microRNAs (total 402 microRNAs) with fold change > 2. The “miRNAs” Venn diagram showed selected microRNAs by threshold of reads per million (RPM) > 1, with 17 microRNAs were downregulated and 28 microRNAs were upregulated in asthmatic bronchial epithelial cells compared to normal human bronchial epithelial cells. The “Targets” Venn diagram showed the predicted genes of microRNAs from “miRNAs” Venn diagram by using miRmap web-based database. The selection threshold was miRmap score ≥ 99.0. The intersection Venn diagram between “Genes” and “Targets” revealed 9 potential microRNA-mRNA interactions.

To identify the potential microRNA-mRNA interactions involved in the homeostasis of asthmatic airway epithelium, we simultaneously analyzed gene expression and microRNA expression by GE microarray and NGS respectively in both normal and asthmatic bronchial epithelial cells (Figure 1). In the GE microarray analysis, we found 227 upregulated genes and 277 downregulated genes with fold change > 2 in asthmatic bronchial epithelial cells compared to normal bronchial epithelial cells. In the NGS data analysis, we found 28 upregulated microRNAs and 17 downregulated microRNAs with fold change > 2 and RPM > 1. The identified microRNAs are shown in Table 1. We attempted to identify whether upregulated (downregulated) genes found in GE microarray are the targets of downregulated (upregulated) microRNAs found in NGS. To predict the putative targets of microRNAs, we used miRmap predictor (http://mirmap.ezlab.org/) and selected the targets with miRmap score > 99.0. Five hundred and twenty six targets of upregulated microRNAs and 578 targets of downregulated microRNAs were harvested from the prediction results. By Venn diagram analysis, 9 genes (2 upregulated and 7 downregulated) were identified with potential microRNA-mRNA interactions in asthmatic bronchial epithelial cells (Table 2).

**Table 1:** MicroRNAs with significant change in asthmatic bronchial epithelial cells versus normal bronchial epithelial cells

#miRNA	Precursor	Asthmatic HBE	Normal HBE	Fold change	Up/down
		Seq (norm)	Seq (norm)		
hsa-miR-190a	hsa-mir-190a	6.06	1.29	4.70	UP
hsa-miR-3065-5p	hsa-mir-3065	24.4	5.49	4.44	UP
hsa-miR-10a-5p	hsa-mir-10a	6937.77	2251.48	3.08	UP
hsa-miR-146a-5p	hsa-mir-146a	157.81	53.57	2.95	UP
hsa-miR-218-5p	hsa-mir-218-1	55.02	18.72	2.94	UP
hsa-miR-218-5p	hsa-mir-218-2	53.98	18.72	2.88	UP
hsa-miR-3935	hsa-mir-3935	11.09	3.87	2.87	UP
hsa-miR-3065-3p	hsa-mir-3065	15.23	5.32	2.86	UP
hsa-miR-486-5p	hsa-mir-486	248.91	93.91	2.65	UP
hsa-miR-19a-3p	hsa-mir-19a	117.14	45.67	2.56	UP
hsa-miR-3661	hsa-mir-3661	5.77	2.26	2.55	UP
hsa-miR-10a-3p	hsa-mir-10a	6.51	2.58	2.52	UP
hsa-miR-19b-3p	hsa-mir-19b-2	409.83	164.11	2.50	UP
hsa-miR-19b-3p	hsa-mir-19b-1	404.95	162.17	2.50	UP
hsa-miR-4521	hsa-mir-4521	12.87	5.16	2.49	UP
hsa-miR-203a	hsa-mir-203a	2852.52	1184.56	2.41	UP
hsa-miR-29c-5p	hsa-mir-29c	9.91	4.36	2.27	UP
hsa-miR-548d-5p	hsa-mir-548d-1	5.47	2.42	2.26	UP
hsa-miR-3613-5p	hsa-mir-3613	3.85	1.77	2.18	UP
hsa-miR-221-5p	hsa-mir-221	415.59	191.86	2.17	UP
hsa-miR-7-5p	hsa-mir-7-2	35.94	16.78	2.14	UP
hsa-miR-7-5p	hsa-mir-7-3	35.94	16.78	2.14	UP
hsa-miR-7-5p	hsa-mir-7-1	36.53	17.1	2.14	UP
hsa-miR-147b	hsa-mir-147b	4.14	1.94	2.13	UP
hsa-miR-140-5p	hsa-mir-140	9.17	4.36	2.10	UP
hsa-miR-548au-5p	hsa-mir-548au	2.37	1.13	2.10	UP
hsa-miR-548o-3p	hsa-mir-548o	27.66	13.23	2.09	UP
hsa-miR-548o-3p	hsa-mir-548o-2	27.66	13.23	2.09	UP
hsa-miR-4517	hsa-mir-4517	3.7	1.77	2.09	UP
hsa-miR-548d-5p	hsa-mir-548d-2	7.69	3.71	2.07	UP
hsa-miR-29b-1-5p	hsa-mir-29b-1	27.66	13.39	2.07	UP
hsa-miR-29a-5p	hsa-mir-29a	11.83	5.81	2.04	UP
hsa-miR-942	hsa-mir-942	9.17	4.52	2.03	UP
hsa-miR-365a-5p	hsa-mir-365a	5.18	2.58	2.01	UP
hsa-miR-122-5p	hsa-mir-122	1.04	2.1	-2.02	Down
hsa-miR-664a-5p	hsa-mir-664a	1.18	2.42	-2.05	Down
hsa-miR-424-3p	hsa-mir-424	127.49	265.6	-2.08	Down
hsa-miR-323a-3p	hsa-mir-323a	1.33	2.9	-2.18	Down
hsa-miR-483-5p	hsa-mir-483	1.48	3.39	-2.29	Down
hsa-miR-24-1-5p	hsa-mir-24-1	1.18	2.74	-2.32	Down
hsa-miR-424-5p	hsa-mir-424	142.13	349.19	-2.46	Down
hsa-miR-665	hsa-mir-665	1.04	2.58	-2.48	Down
hsa-miR-487a	hsa-mir-487a	1.04	2.74	-2.63	Down
hsa-miR-33b-5p	hsa-mir-33b	12.87	34.53	-2.68	Down
hsa-miR-4792	hsa-mir-4792	6.95	19.69	-2.83	Down
hsa-miR-548h-5p	hsa-mir-548h-1	1.18	3.55	-3.01	Down
hsa-miR-548h-5p	hsa-mir-548h-2	1.18	3.55	-3.01	Down
hsa-miR-548h-5p	hsa-mir-548h-3	1.18	3.55	-3.01	Down
hsa-miR-548h-5p	hsa-mir-548h-4	1.18	3.55	-3.01	Down
hsa-miR-548h-5p	hsa-mir-548h-5	1.18	3.55	-3.01	Down
hsa-miR-3177-3p	hsa-mir-3177	1.04	3.39	-3.26	Down
hsa-miR-212-3p	hsa-mir-212	1.48	5.32	-3.59	Down
hsa-miR-873-5p	hsa-mir-873	1.63	9.84	-6.04	Down
hsa-miR-3176	hsa-mir-3176	5.18	39.37	-7.60	Down
hsa-miR-6499-5p	hsa-mir-6499	5.62	78.74	-14.01	Down

**Table 2:** Genes selected by intersection between GE array candidates and microRNA putative targets

Official gene symbol	Gene name	Species	Fold change (Asthma/normal)	Expression
*FGF2*	fibroblast growth factor 2 (FGF2)	Homo sapiens	2.08	Up
*IRAK3*	interleukin 1 receptor associated kinase 3 (IRAK3)	Homo sapiens	0.48	Down
*LOX*	lysyl oxidase (LOX)	Homo sapiens	0.13	Down
*MDGA1*	MAM domain containing glycosylphosphatidylinositol anchor 1 (MDGA1)	Homo sapiens	0.21	Down
*MEF2C*	myocyte enhancer factor 2C (MEF2C)	Homo sapiens	0.48	Down
*NAIP*	NLR family apoptosis inhibitory protein (NAIP)	Homo sapiens	0.3	Down
*TFPI*	tissue factor pathway inhibitor (TFPI)	Homo sapiens	0.42	Down
*TP53I11*	tumor protein p53 inducible protein 11 (TP53I11)	Homo sapiens	3.52	Up
*NEURL1*	neuralized E3 ubiquitin protein ligase 1 (NEURL1)	Homo sapiens	0.38	Down

### Downregulation of *MEF2C* and *MDGA1* may be involved in the homeostasis of asthmatic airway epithelium

**Figure 2:**
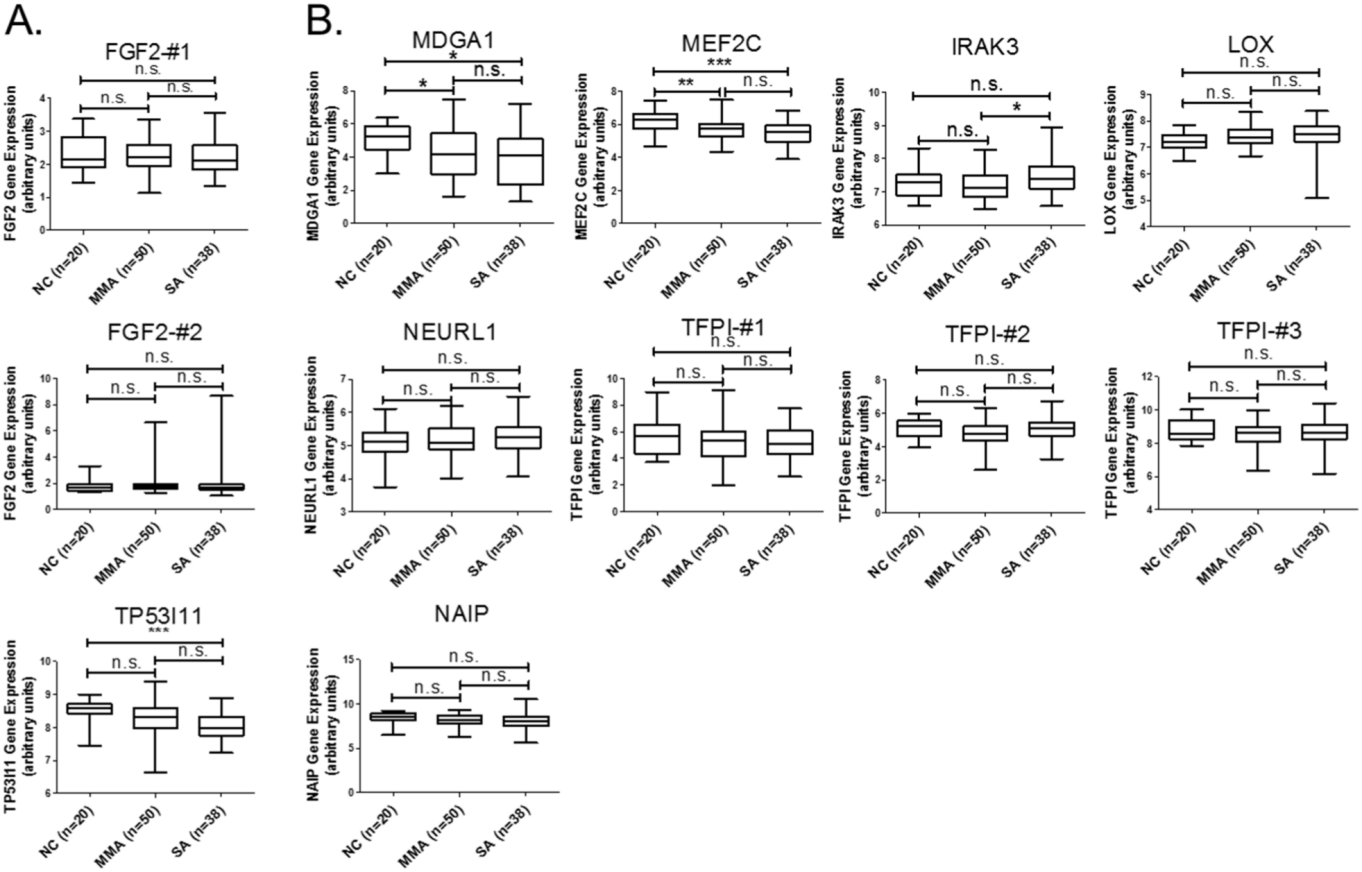
GEO database analysis of 9 genes with potential microRNA-mRNA interactions Gene expressions of the **(A)** upregulated and **(B)** downregulated genes shown in Table 2 were analyzed using GSE43696 microarray data from the GEO database. The results showed that gene expressions of both *MEF2C* and *MDGA1* were significantly downregulated in patients with either mild-moderate or severe asthma compared to normal controls. n.s. means no significance, * represents p-value < 0.05, ** represents p-value < 0.01, and *** represents p-value < 0.001. (Probe information of GSE43696 array from GEO database: FGF2-#1, A_23_P218918; FGF2-#2, A_24_P931472; TP53I11, A_23_P368028; MDGA1, A_23_P310460; MEF2C, A_23_P320739; IRAK3, A_23_P162300; LOX, A_23_P122216; NEURL1-#1, A_23_P138492; TFPI-#1, A_23_P156826; TFPI-#2, A_23_P330070; TFPI-#3, A_23_P17095; NAIP, A_23_P110473.)

We next analyzed the functionally related pathways of these 9 genes to explore the possible mechanisms underlying epithelial dysfunction in asthma. We used KEGG pathways of DAVID database (https://david.ncifcrf.gov/) and set the criteria as EASE = 1. The result indicated that 2 genes, *FGF2* (upregulated, Figure 2A) and *MEF2C* (downregulated, Figure 2B), were involved in the MAPK pathway (Table 3). To apply the identified 9 candidates to more clinical asthma samples, we used GEO bioinformatics database (https://www.ncbi.nlm.nih.gov/geo/) and selected a representative microarray (accession number, GSE43696) that contains bronchial epithelial cells form 50 patients with mild-moderate asthma, 38 patients with severe asthma, and 20 normal controls. The probe set in our microarray data was not completely identical to the probe set of GSE43696 array we analyzed in GEO database. The “#” represents that the probe is different and the number represents that there are more than one probe for a gene. We analyzed the expression patterns of genes with identical probes as in our GE array library. For few genes without any identical probes, we still analyzed their gene expression patterns and marked them with “#-number” in the figures. The results showed that gene expressions of both *MEF2C* and *MDGA1* were significantly downregulated in patients with either mild-moderate or severe asthma compared to normal controls (Figure 2B). *MEF2C* and *MDGA1* were the targets of mir-203a and mir-3605-3p, respectively (Table 4). The sequence alignment of mir-203a in the 3’UTR of *MEF2C* and mir-3065-3p in the 3’UTR of *MDGA1* was analyzed by 3 microRNA target prediction databases, including miRmap, TargetScan, and miRDB. The sequence conservation analysis indicated that the position of 1331-1337 of *MEF2C* 3’UTR was the putative binding site for mir-203a (Supplementary Table 1; Supplementary Figure 1), and the position of 1515-1521 of *MDGA1* 3’UTR is the putative binding site for mir-3065-3p (Supplementary Table 2; Supplementary Figure 2). These data suggested that regulation of mir-203a to *MEF2C* and mir-3065-3p to *MDGA1* may play important roles in the airway epithelial homeostasis in asthma.

**Table 3:** KEGG pathway analysis of 9 genes with potential microRNA-mRNA interaction

KEGG pathway	Count	P-value	Genes up	Genes down	Fold enrichment
MAPK signaling pathway	2	1.40E-01	*FGF2*	*MEF2C*	10.84

**Table 4:** MicroRNAs and the corresponding predicted targets

miRNA	Precursor	Asthmatic HBE	Normal HBE	Fold change	Log2 (ratio)	Predicted targets
		Seq (norm)	Seq (norm)			
Hsa-miR-203a	hsa-mir-203a	2852.52	1184.56	2.41	1.27	*MEF2C*
hsa-miR-3065-3p	hsa-miR-3065	15.23	5.32	2.86	1.52	*MDGA1*

### Downregulation of *KCNJ2* may be associated with severity of asthma

**Figure 3:**
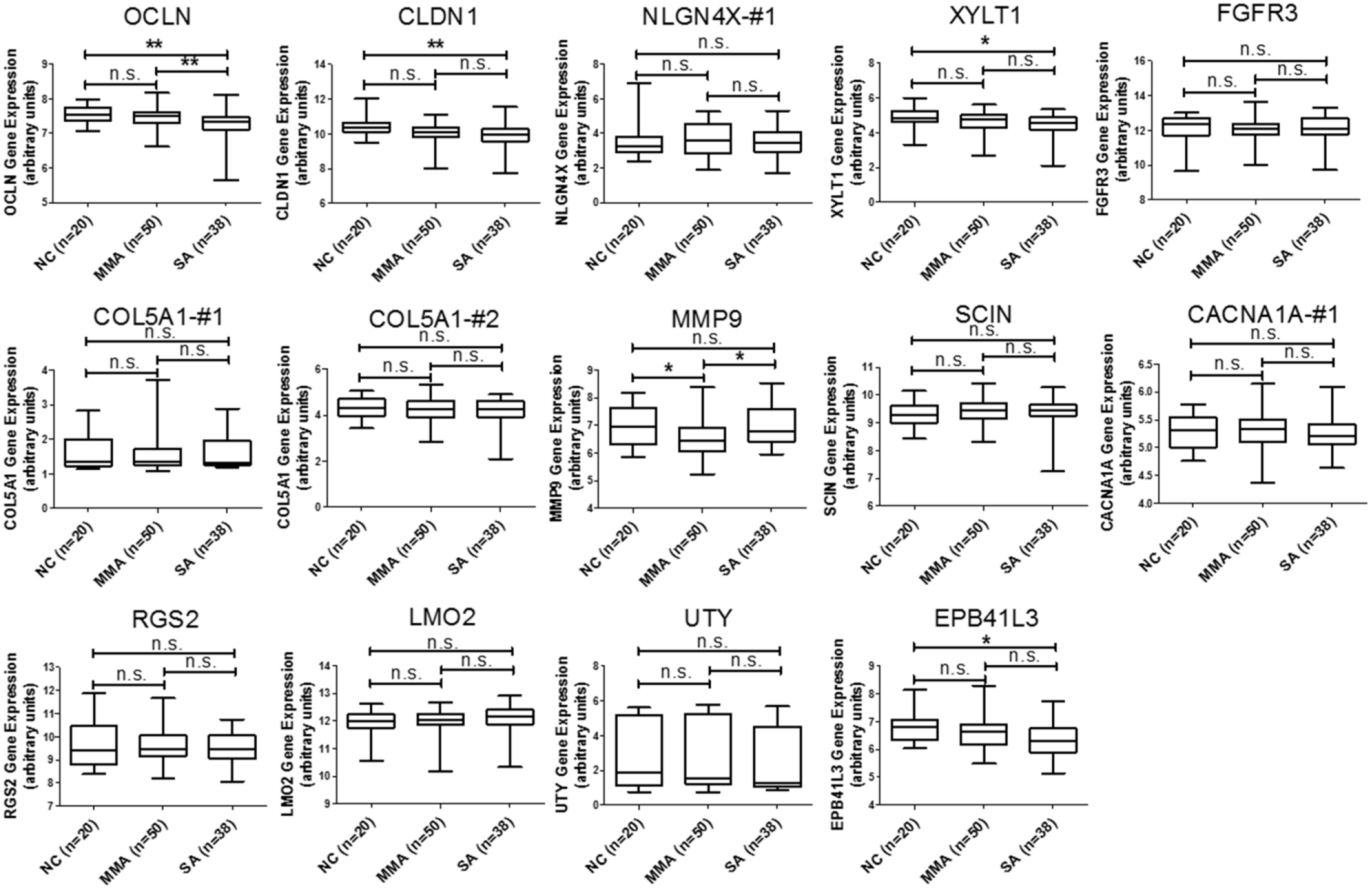
GEO database analysis of upregulated genes identified from KEGG pathway analysis Gene expressions of the 13 upregulated genes shown in Table 5 were analyzed using GSE43696 microarray data from the GEO database. None of these 13 genes were significantly upregulated in patients with asthma. n.s. means no significance, * represents p-value < 0.05, ** represents p-value < 0.01, and *** represents p-value < 0.001. (Probe information of GSE43696 array from GEO database: OCLN, A_23_P92672; CLDN1, A_23_P57784; NLGN4X-#1, A_23_P364592; XYLT1, A_24_P787897; FGFR3, A_23_P500501; COL5A1-#1, A_23_P158593; COL5A1-#2, A_23_P83818; MMP9, A_23_P40174; SCIN, A_23_P157136; CACNA1A-#1, A_24_P130559; RGS2, A_23_P114947; LMO2, A_23_P53126; UTY, A_23_P329835; EPB41L3, A_23_P4536.)

**Figure 4:**
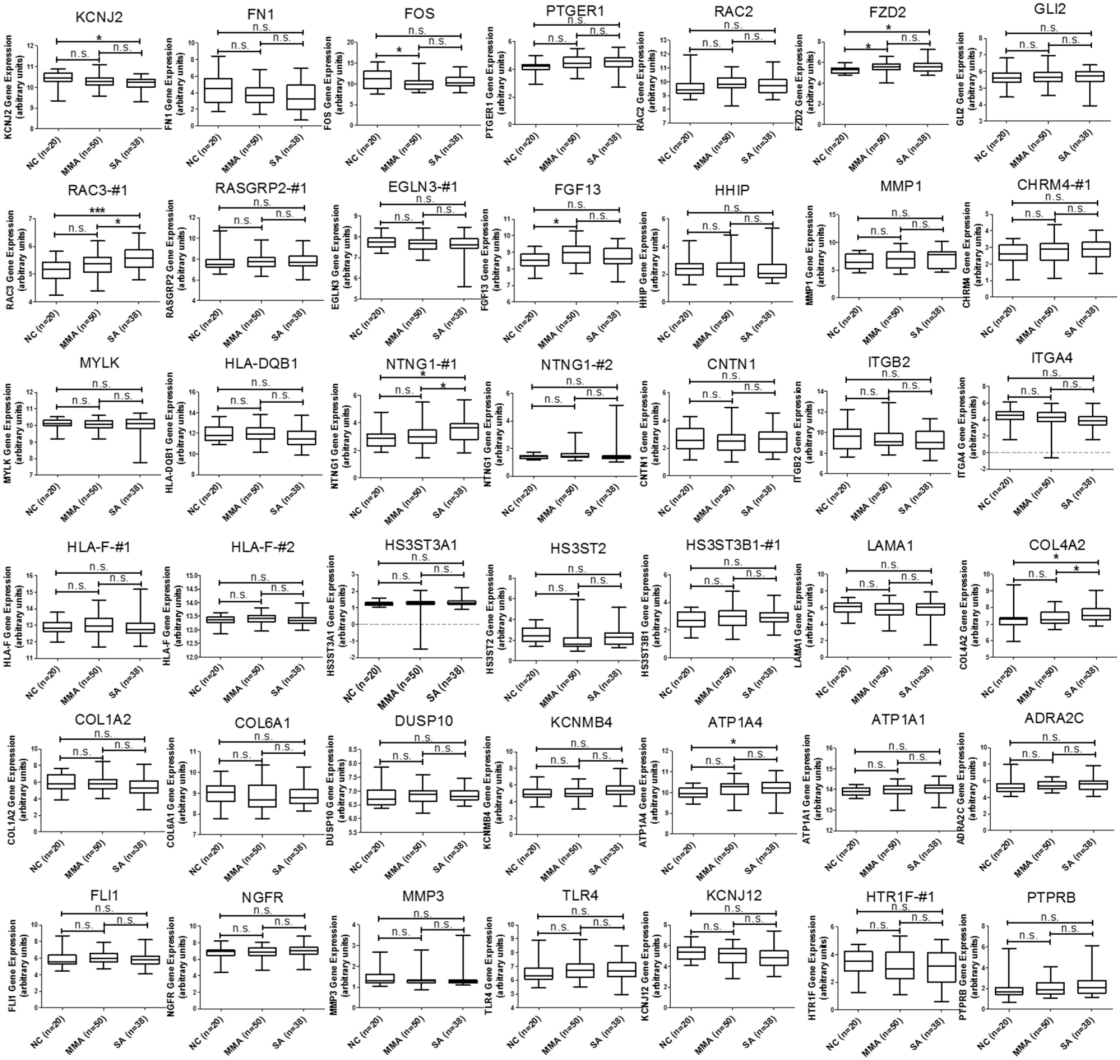
GEO database analysis of downregulated genes identified from KEGG pathway analysis Gene expressions of the 40 downregulated genes shown in Table 5 were analyzed using GSE43696 microarray data from the GEO database. The results showed that *KCNJ2* expression was significantly downregulated in patients with severe asthma. n.s. means no significance, * represents p-value < 0.05, ** represents p-value < 0.01, and *** represents p-value < 0.001. (Probe information of GSE43696 array from GEO database: KCNJ2, A_23_P329261; FN1, A_24_P334130; FOS, A_23_P106194; PTGER1, A_23_P4808; RAC2, A_ 23_P218770 ; FZD2, A_23_P141362; GLI2, A_23_P209246; RAC3-#1, A_23_P125001; RASGRP2-#1, A_23_P64058; EGLN3-#1, A_23_P360379; FGF13, A_23_P217319; HHIP, A_23_P167129; MMP1, A_23_P1691; CHRM4-#1, A_23_P104845; MYLK, A_23_P143817; HLA-DQB1, A_23_P8108; NTNG1-#1, A_23_P201547; NTNG1-#12, A_24_P359671; CNTN1, A_23_P390700; ITGB2, A_23_P329573; ITGA4, A_23_P56505; HLA-F-#1, A_23_P145264; HLA-F-#2, A_23_P145264; HS3ST3A1, A_23_P66525; HS3ST2, A_23_P118158; HS3ST3B1-#1, A_23_P77918; LAMA1, A_32_P313405; COL4A2, A_23_P205031; COL1A2, A_24_P277934; COL6A1, A_32_P32254; DUSP10, A_24_P182494; KCNMB4, A_23_P64792; ATP1A4, A_23_P160177; ATP1A1, A_23_P1072; ADRA2C, A_23_P256158; FLI1, A_24_P355649; NGFR, A_23_P389897; MMP3, A_23_P161698; TLR4, A_24_P69538; KCNJ12, A_24_P339429; ITGA4, A_23_P56505; HTR1F-#1, A_23_P166674; PTPRB, A_23_P53390.)

In addition to the analysis of microRNA-mRNA interaction, we also intended to identify novel genes involved in the homeostasis of asthmatic airway epithelium. We mapped the 227 upregulated and 277 downregulated genes discovered from our GE microarray data to KEGG pathways and identified 13 upregulated and 40 downregulated genes related to 14 KEGG pathways, which were associated with cell adhesion, ECM-receptor interaction, focal adhesion, adherens junctions, tight junction, and MAPK signaling pathway (Table 5). To demonstrate the significance of these differentially expressed genes in asthmatic bronchial epithelium, we first analyzed gene expression pattern of the 13 upregulated genes (Table 5, Genes Up) using the GSE43696 microarray data. We found none of them were significantly upregulated in patients with asthma (Figure 3). We next analyzed the gene expression pattern of the 40 downregulated genes (Table 5, Genes Down). The results showed that *KCNJ2* expression was significantly downregulated in patients with severe asthma (Figure 4).

**Table 5:** KEGG pathway analysis of dysregulated genes identified from GE microarray

KEGG pathways	Count	P-value	Genes up	Genes down	Fold enrichment
Cell adhesion molecules (CAMs)	10	1.08E-02	*OCLN, NLGN4X, CLDN1*	*HLA-DQB1, NTNG1, CNTN1, ITGB2, ITGA4, CLDN20, HLA-F*	2.72
Glycosaminoglycan biosynthesis - heparan sulfate / heparin	4	2.29E-02	*XYLT1*	*HS3ST3A1, HS3ST2, HS3ST3B1*	6.43
ECM-receptor interaction	7	2.43E-02	*COL5A1*	*LAMA1, COL4A2, COL1A2, COL6A1, ITGA4, FN1*	3.11
Pathways in cancer	18	2.43E-02	*FZD10, FGFR3, MMP9, FGF2*	*LAMA1, FOS, PTGER1, COL4A2, RAC2, RAC3, RASGRP2, EGLN3, FGF13, HHIP, FZD2, GLI2, MMP1, FN1*	1.77
Regulation of actin cytoskeleton	11	4.55E-02	*FGFR3, SCIN, FGF2*	*CHRM4, RAC2, RAC3, FGF13, ITGB2, ITGA4, MYLK, FN1*	2.01
Focal adhesion	10	8.41E-02	*COL5A1*	*LAMA1, COL4A2, RAC2, RAC3, COL1A2, COL6A1, ITGA4, MYLK, FN1*	1.87
MAPK signaling pathway	10	2.11E-01	*FGFR3, FGF2, CACNA1A*	*MEF2C, FOS, RAC2, RAC3, RASGRP2, DUSP10, FGF13*	1.51
cGMP-PKG signaling pathway	7	2.56E-01	*RGS2*	*MEF2C, KCNMB4, ATP1A4, ATP1A1, ADRA2C, MYLK*	1.63
Transcriptional misregulation in cancer	7	2.65E-01	*LMO2, UTY, MMP9*	*MEF2C, FLI1, NGFR, MMP3*	1.61
PI3K-Akt signaling pathway	12	2.75E-01	*FGFR3, FGF2, COL5A1*	*LAMA1, COL4A2, COL1A2, COL6A1, FGF13, TLR4, NGFR, ITGA4, FN1*	1.34
Oxytocin signaling pathway	6	3.85E-01	*RGS2*	*MEF2C, FOS, KCNJ12, KCNJ2, MYLK*	1.47
cAMP signaling pathway	7	4.01E-01		*FOS, RAC2, RAC3, ATP1A4, ATP1A1, HHIP, HTR1F*	1.36
Adherens junction	3	5.50E-01		*PTPRB, RAC2, RAC3*	1.63
Tight junction	4	6.91E-01	*EPB41L3, OCLN, CLDN1*	*CLDN20*	1.13

### The differentially expressed genes in asthmatic bronchial epithelial cells were correlated with cell adhesion in functional annotation analysis

**Figure 5:**
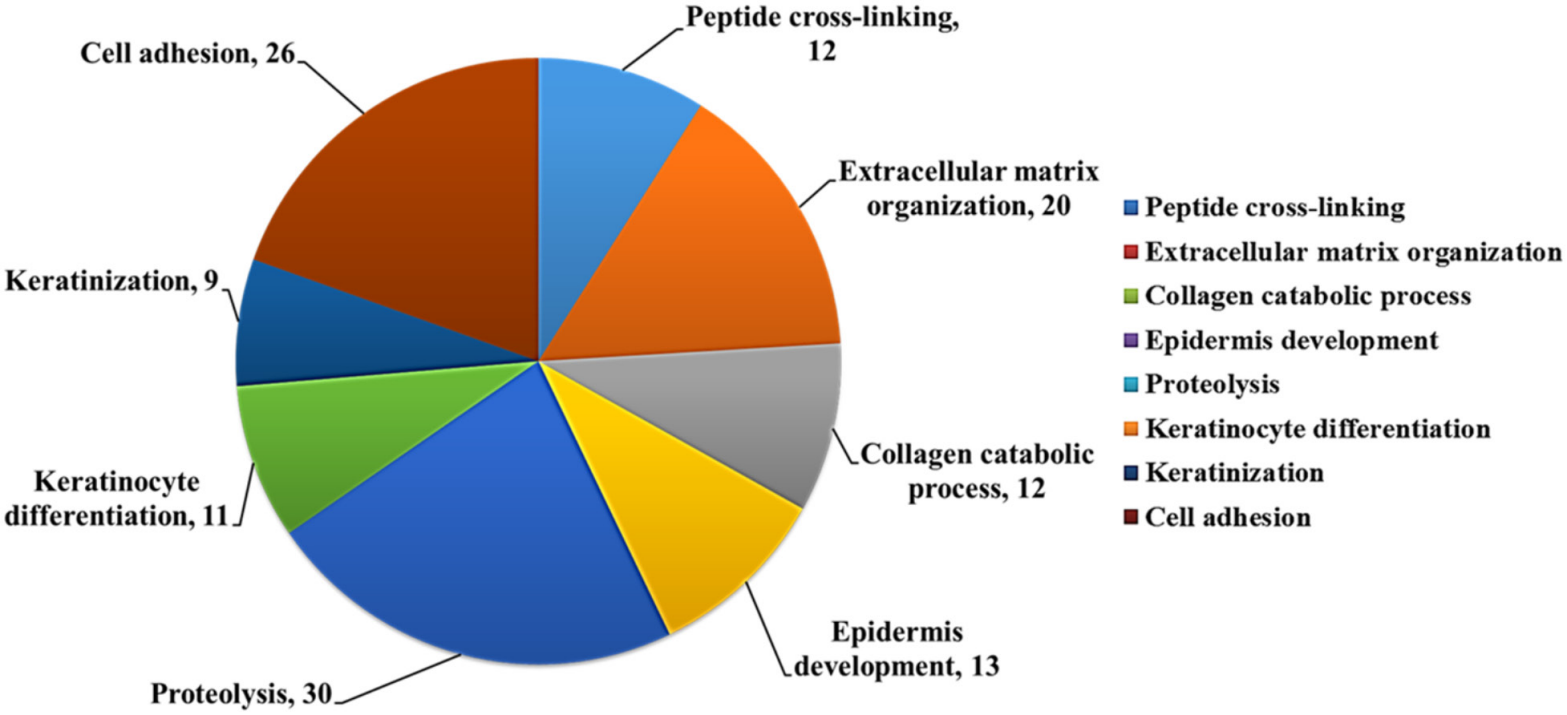
Biological function analysis of differentially expressed genes in asthmatic bronchial epithelial cells Functional analysis of 504 differentially expressed genes discovered from the GE microarray data was performed by functional annotation (Biological Processes) in DAVID database. The results showed that these genes were involved in cell adhesion (26 genes), keratinization (9 genes), keratinocyte differentiation (11 genes), proteolysis (30 genes), collagen catabolic process (12 genes), extracellular matrix organization (20 genes), and peptide cross-linking (12 genes). The selected criteria were EASE = 0.1, p-value < 0.05, and fold enrichment > 1.3.

We used DAVID database to analyze potential biological function of the differentially expressed genes (227 upregulated and 277 downregulated) in asthmatic bronchial epithelial cells. The functional annotation analysis showed that these genes were involved in cell adhesion (26 genes), keratinization (9 genes), keratinocyte differentiation (11 genes), proteolysis (30 genes), collagen catabolic process (12 genes), extracellular matrix organization (20 genes), and peptide cross-linking (12 genes) (Figure 5).

## DISCUSSION

Bronchial epithelial cells play as a front-line defense barrier against environmental stimuli. The cell-environment interaction results in activation of complex biological processes and contributes to the progression and exacerbation of asthma. A detailed understanding of gene regulations in airway epithelial cells may provide new insights into pathogenesis and potential therapeutic implications in asthma. In this study, we analyzed genetic expression profiles of mRNAs (GE array) and microRNAs (NGS) in normal and asthmatic bronchial epithelial cells, and explored the potential molecular mechanisms of gene regulation using bioinformatics approaches. By using miRmap for targets prediction and Venn diagram for intersection analysis, we first identified 9 genes that potentially involved in the microRNA-mRNA interactions in asthma. We further analyzed these candidate genes in the GEO database using a representative microarray (GSE43696) and found 2 potential microRNA-mRNA interactions, of which mir-203a may repress *MEF2C* and mir-3065-3p may repress *MDGA1*. The expressions of both mir-203a and mir-3065-3p were upregulated in asthmatic bronchial epithelial cells and the expressions of *MEF2C* and *MDGA1* were downregulated.

Mir-203a has been reported as a tumor suppressor in many cancers, by suppressing cell proliferation, migration, and invasion [38, 39]. It was also shown to promote apoptosis in lung cancer cells [40]. The expression of mir-203a was downregulated in many cancers, suggesting its important role of growth inhibition [41, 42]. We found that asthmatic bronchial epithelial cells expressed higher mir-203a than normal healthy bronchial epithelial cells, which implicated its effects to negatively regulate cell proliferation. *MEF2C* (myocyte enhancer factor 2C), identified as the putative target of mir-203a, was downregulated in asthmatic bronchial epithelial cells in our analysis. *MEF2C* is a transcription factor which is involved in MAPK signaling pathway to regulate B-cell proliferation [43]. High expression of *MEF2C* was found in lymphoblastic leukemia [44]. Overexpression of *MEF2C* can regulate hepatocellular carcinoma progression through endothelial growth factor and wnt/beta-catenin signaling pathway [45, 46]. These reports suggested that expression of *MEF2C* may play as a positive regulator in cell growth. We postulated a novel microRNA-mRNA interaction in asthma that the upregulated mir-203a may target *MEF2C*, leading to the decreased cell proliferation in asthmatic bronchial epithelial cells.

There were little reports demonstrating functions of mir-3065-3p, but it has been found to be upregulated in breast cancer cells under hypoxia treatment (16, 32, and 48h) [47]. Hypoxia can cause accumulated oxidative stress and thus induce apoptosis [48]. Thus, the expression of mir-3065-3p may also be involved in cell proliferation. Here, we speculated the expression of mir-3065-3p may regulate cell adhesion because its putative target gene *MDGA1* (MAM domain containing glycosylphosphatidylinositol anchor 1) has been shown to be localized in lipid raft and involved in cell-cell adhesion [49, 50]. We thought that upregulation of mir-3065-3p can repress *MDGA1* expression and suppress the cell-cell adhesion in asthmatic bronchial epithelial cells.

*KCNJ2* (potassium voltage-gated channel subfamily J member 2) regulates K^+^ to flow into cells in response to various biological functions. The aberrant homeostasis of K^+^ concentration can affect cell proliferation [51]. Low concentration of intracellular K^+^ was shown to promote apoptosis [52–54]. Inhibition of *KCNJ2* can increase the cisplatin-induced apoptosis in oral cancer [55]. The importance of K^+^ channel in normal HBE cells was demonstrated [56]. We found that *KCNJ2* was downregulated in asthmatic bronchial epithelial cells, which may lead to low intracellular K^+^ level and therefore promote cells apoptosis. From the analysis of GEO database, expression of *KCNJ2* is decreased in patients with severe asthma, suggesting its expression may be associated with mechanisms of airway remodeling driving by epithelial dysfunction.

**Figure 6:**
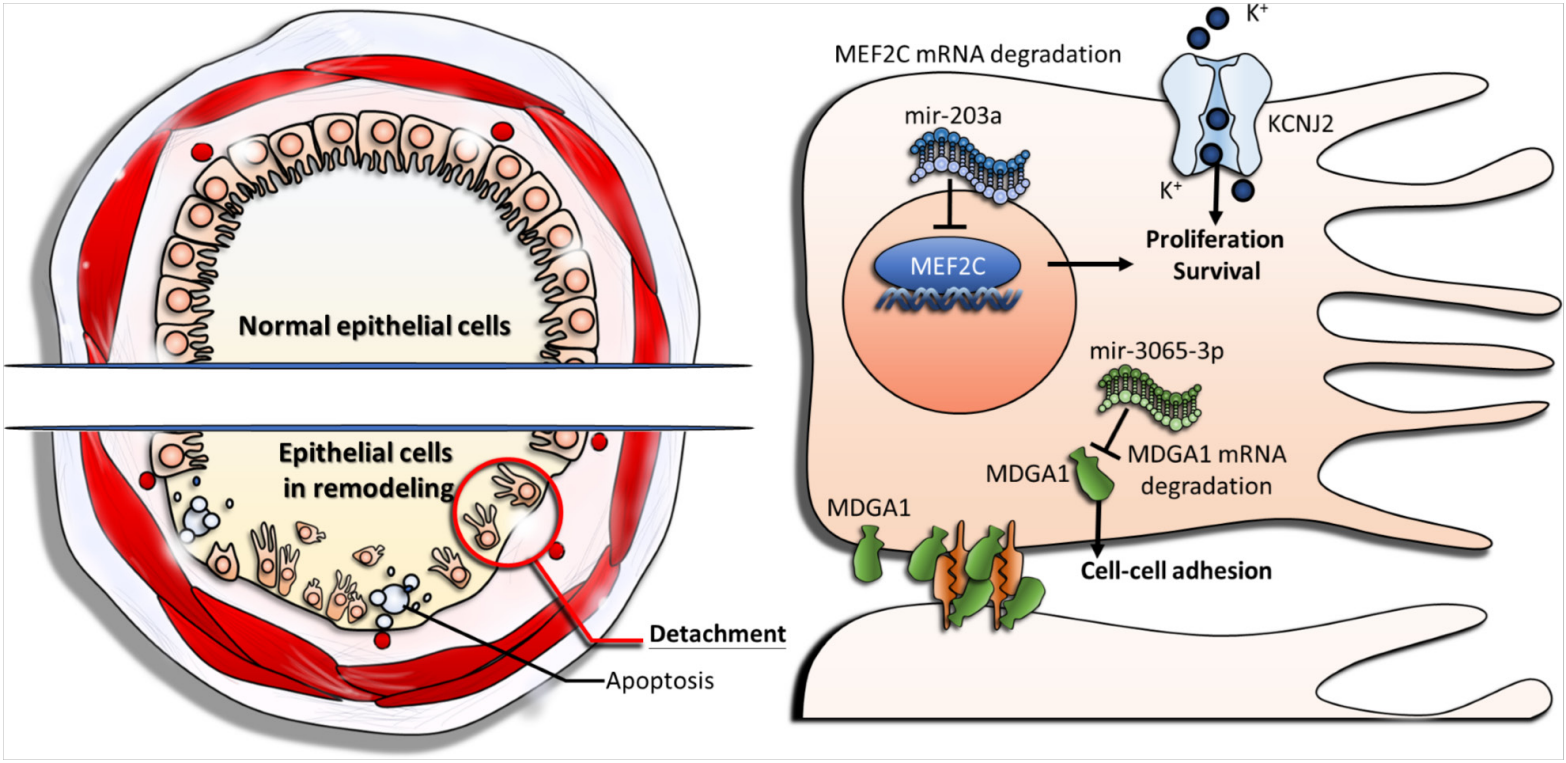
The proposed novel molecular mechanisms of gene regulations involved in airway epithelial homeostasis of asthma Upregulation of mir-203a represses *MEF2C* expression and leads to suppression of cell proliferation. Upregulation of mir-3065-3p represses *MDGA1* expression and causes decreased cell-cell adhesion. Downregulation of *KCNJ2* alters potassium homeostasis, results in low intracellular potassium concentration, and therefore promote apoptosis of epithelial cells.

It is known that airway remodeling is associated with clinical symptoms and progression of asthma by its irreversible structure changes in airway wall, and damage of the bronchial epithelium play a key role in driving airway remodeling [10]. The representative features were characterized by epithelial cell death and detachment (shedding). Our study was carried out using a combination of microarray, NGS, and high-throughput analysis of bioinformatics. We identified novel genetic regulations associated with proliferation, apoptosis, and cell-cell adhesion of asthmatic bronchial epithelial cells (Figure 6). In conclusion, our study shows that aberrant regulations of mir-203a to *MEF2C* and mir-3065-3p to *MDGA1*, as well as downregulation of *KCNJ2*, play important roles in airway epithelial homeostasis in asthma. These findings provide new perspectives on the development of diagnostic or therapeutic strategies targeting bronchial epithelium for asthma. The approach in this study also provides a new aspect of studying the pathogenesis of asthma.

## MATERIALS AND METHODS

### Primary cells

Primary normal human bronchial epithelial cells (NHBE) and asthmatic bronchial epithelial cells (DHBE) were purchased from Lonza Walkersville, Inc. The cell culture was performed following the manufacturer’s protocol. Cells were grown in Bronchial Epithelial Cell Basal Media (BEGM Bulletkit™; Lonza, Clonetics, San Diego, CA) supplemented with 2 ml BPE, 0.5 ml hydrocortisone, 0.5 ml hEGF, 0.5 ml epinephrine, 0.5 ml transferrin, 0.5 ml insulin, 0.5 ml retinoic acid, 0.5 ml triiodothyronine, and 0.5 ml GA-1000. Cells were maintained at 37°C in 5% CO_2_ incubator, and passaged with ReagentPack™ (Lonza; Clonetics, San Diego, CA, USA) containing trypsin-EDTA, trypsin neutralizing solution, and HEPES buffered solution.

### Next-generation sequencing (NGS)

The expression profile of microRNAs was examined by using NGS [29]. Total RNA of both normal and asthmatic bronchial epithelial cells was extracted by using Trizol^®^ Reagent (Invitrogen, USA) according to manufacturer’s instructions. The quality of OD260 nm was detected by using a ND-1000 spectrophotometer (Nanodrop Technology, USA). Samples were applied to Welgene Biotechnology Company (Welgene, Taipei, Taiwan) for RNA preparation and sequencing analysis. The integrity and concentration of RNA samples were assessed by a Bioanalyzer 2100 (Agilent Technology, USA) with RNA 6000 LabChip kit (Agilent Technologies, USA). To construct the small RNA library and perform deep sequencing, samples were prepared using Illumina sample preparation kit according to the TruSeq Small RNA Sample Preparation Guide. The total RNA was ligated with 3′ and 5′ adaptors and reverse-transcribed into cDNA by PCR amplification. The harvested cDNA constructs were fractionated by size on a 6% polyacrylamide gel electrophoresis and the bands containing 18-40 nucleotide RNA fragments (140-155 nucleotide in length with both adapters) were purified. Libraries were then sequenced on an Illumina GAIIx instrument (50 cycle single read) and the sequencing results were processed with the Illumina software. To analyze small RNA sequencing, the sequences were applied to go through a filtering process to obtain qualified reads. ConDeTri [57] was used to trim or remove the reads according to the quality score. The qualified reads were then analyzed using miRDeep2 [58] to clip the 3′ adapter sequence and remove shorter reads (< 18 nucleotides), before aligning reads to the human genome from UCSC. Because miRNAs are usually mapped to few genomic locations, only reads mapped perfectly to the genome five or less times were used for miRNA detection. MiRDeep2 was used to estimate expression levels of miRNAs. The criteria for microRNAs selection were fold change > 2, and reads per million (RPM) > 1.

### Microarray analysis

Total RNA of bronchial epithelial cells was extracted by using Trizol^®^ Reagent (Invitrogen, USA) according to manufacturer’s instructions. The quality of OD260 nm was detected by using a ND-1000 spectrophotometer (Nanodrop Technology, USA). Samples were applied to Welgene Biotechnology Company (Welgene, Taipei, Taiwan) for RNA preparation and microarray analysis. The detailed protocol was described previously [59, 60].

### miRmap database analysis

miRmap is an open-source software library providing comprehensive microRNA targets prediction (http://mirmap.ezlab.org/) [35]. The putative target genes can be identified based on calculating the complementary ability of microRNA-mRNA interactions. The predictor also estimates the strength of mRNA repression for ranking potential candidate targets by employing few features, including thermodynamic, evolutionary, probabilistic or sequence-based features. The prediction results provide a list of putative target genes with miRmap score, which is a predictive reference value. We selected putative microRNA targets with miRmap score ≥ 99.0 in this study.

### DAVID database analysis

The Database for Annotation, Visualization and Integrated Discovery (DAVID) is a powerful gene functional classification tool that integrates multiple functional annotation databases, including Gene Ontology (GO), Biological process, or KEGG pathway (https://david.ncifcrf.gov/) [36]. A list of interesting genes can be classified into clusters of related biological functions, signaling pathways, or diseases by calculating the similarity of global annotation profiles with agglomeration algorithm method. It also provides the criteria of EASE score for analysis, which is a modified Fisher’s Exact P-value. The reference score represents how specifically the user genes are involved in the category (for example: signaling pathways). We selected EASE score = 0.1 as default and 1 to extend clustering range in our analysis.

### Gene expression omnibus (GEO) database analysis

GEO is a web-database which collects submitted high throughput gene expression data of microarray, chips, or NGS (https://www.ncbi.nlm.nih.gov/geo/) [37]. The microarray of accession number GSE43696 published in 2014 [61] was used in this study. This microarray contains gene expression information of clinical bronchial epithelial cells from 108 patients, including normal control (n = 20, 9 males and 11 females), mild-moderate asthma (n = 50, 15 males and 35 females), and severe asthma (n = 38, 10 males and 28 females). The raw data were extracted and re-plotted by GraphPad prism 5 software (GraphPad Software, La Jolla, CA, USA).

### Statistical analysis

The raw data extracted from GEO database were statistically analyzed using *one-way ANOVA with Tukey post hoc test* by GraphPad Prism 5 software (GraphPad Software, La Jolla, CA, USA).

## SUPPLEMENTARY MATERIALS FIGURES AND TABLES


