# Supplementary material for: Identification of novel genetic regulations associated with airway epithelial homeostasis using next-generation sequencing data and bioinformatics approaches
Source: Oncotarget. 2017 Jul 31;8(47):82674–88. doi: 10.18632/oncotarget.19752 (PMC5669919; doi:10.18632/oncotarget.19752)
Supplement: Supplementary file 1 [file oncotarget-08-82674-s001.pdf]

Identification of novel genetic regulations associated with airway epithelial homeostasis using next-generation sequencing data and bioinformatics approaches

SUPPLEMENTARY MATERIALS

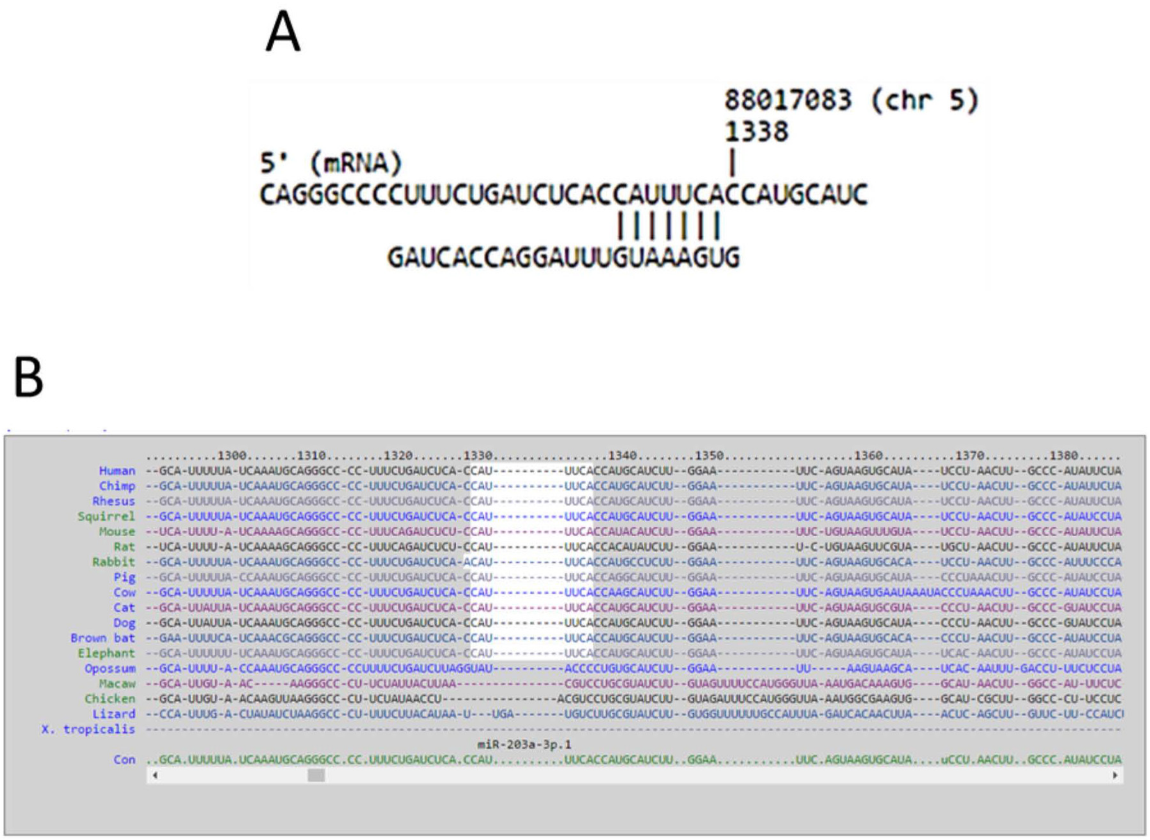

Supplementary Figure 1: The position of 1331-1337 of *MEF2C* 3'UTR was the putative binding site for mir-203a. **(A)** The sequence alignment of mir-203a in the 3'UTR of *MEF2C* and **(B)** the sequence conservation comparison in different species.

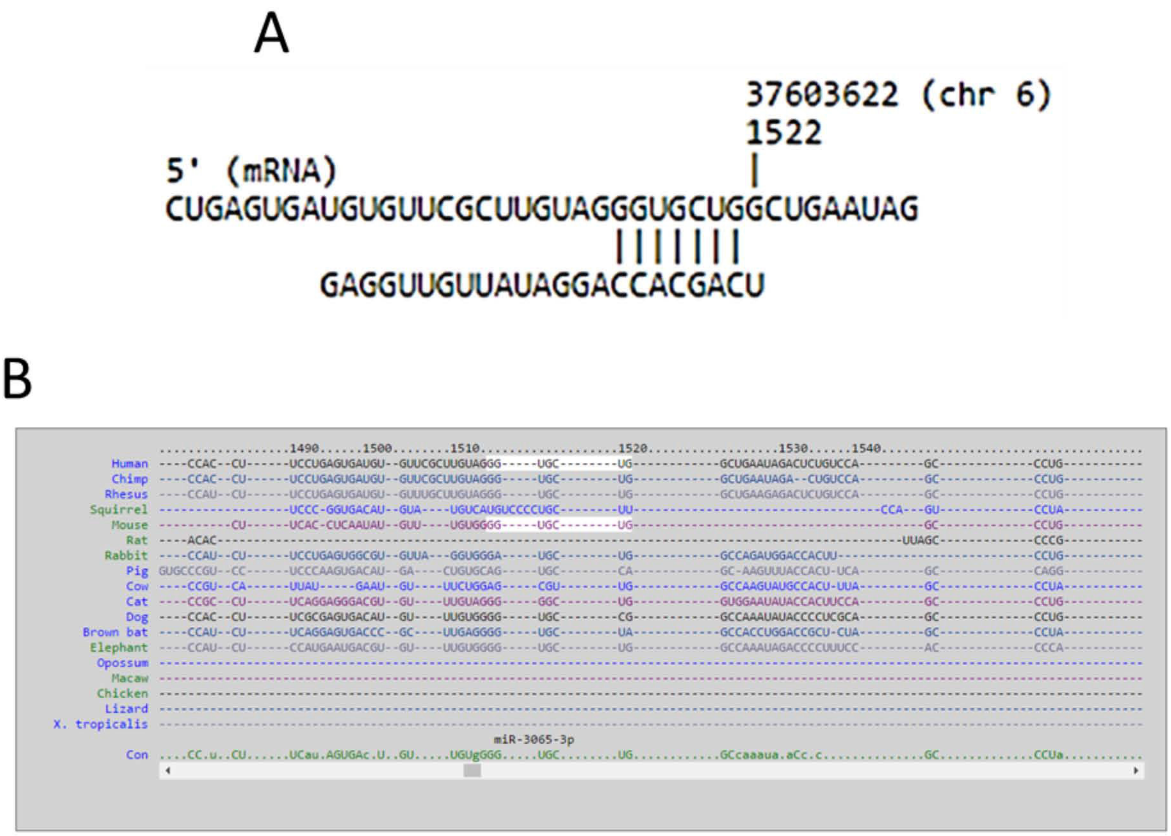

Supplementary Figure 2: The position of 1515-1521 of *MDGA1* 3'UTR is the putative binding site for mir-3065-3p. **(A)** The sequence alignment of mir-3065-3p in the 3'UTR of *MDGA1* and **(B)** the sequence conservation comparison in different species.

Supplementary Table 1: Summary of mir-203a targeting *MEF2C*

Prediction of mir-203a targeting sites on MEF2C										
miRmap		+	+	+		+	+	+	+	
TargetScan		+	+		+	+	+	+		+
miRDB		+	+			+	+	+		
Binding sites (3'UTR)		1331<br>I<br>1337	1759<br>I<br>1765	1912<br>I<br>1917	2044<br>I<br>2050	2277<br>I<br>2283	2487<br>I<br>2494	2509<br>I<br>2515	3251<br>I<br>3256	5693<br>I<br>5699
3'UTR sequence conservation in species	Human	+	+	+	+	+	+	+	+	+
	Chimp	+	+	+	+	+		+	+	+
	Rhesus	+	+	+	+	+		+	+	+
	Squirrel	+	+	+	+	+		+	+	
	Mouse	+	+	+		+			+	
	Rat	+	+			+		+		
	Rabbit	+	+		+	+			+	
	Pig	+	+	+	+	+		+	+	
	Cow	+	+	+	+	+		+	+	
	Cat	+		+	+			+	+	
	Dog	+	+	+	+			+	+	
	Brown bat	+	+	+		+		+	+	
	Elephant	+				+		+	+	
	Opossum							+	+	
	Macaw			+					+	
	Chicken							+	+	
	Lizard									

"+" means the position identified in the databases and identical in species.

Supplementary Table 2: Summary of mir-3065-3p targeting *MDGA1*

Prediction of mir-3065-3p targeting sites on *MDGA1*							
miRmap		+	+	+	+	+	+
TargetScan		+		+	+	+	
miRDB		+		+	+	+	
Binding sites (3'UTR)		1515<br>I<br>1521	2817<br>I<br>2822	3133<br>I<br>3139	3194<br>I<br>3200	3265<br>I<br>3272	4111<br>I<br>4116
3'UTR sequence conservation in species	Human	+	+	+	+	+	+
	Chimp		+				+
	Rhesus		+				+
	Squirrel						+
	Mouse	+					
	Rat						
	Rabbit						
	Pig						+
	Cow						+
	Cat						+
	Dog						+
	Brown bat						
	Elephant						+
	Opossum						
	Macaw						
	Chicken						
	Lizard						

"+" means the position identified in the databases and identical in species.
